# Retrospective Study of Dupilumab Treatment for Moderate to Severe Atopic Dermatitis in Korea: Efficacy and Safety of Dupilumab in Real-World Practice

**DOI:** 10.3390/jcm9061982

**Published:** 2020-06-24

**Authors:** Dong Hyek Jang, Seok Jae Heo, Hye Jung Jung, Mi Yeon Park, Seong Jun Seo, Jiyoung Ahn

**Affiliations:** 1 Department of Dermatology, National Medical Center, Seoul 04564, Korea; jangdh321@nmc.or.kr (D.H.J.); humeong01@nmc.or.kr (H.J.J.); miyeon.park@nmc.or.kr (M.Y.P.); 2 Department of Biostatistics and Computing, Yonsei University Graduate School, Seoul 03722, Korea; lbthinking91@gmail.com; 3 Department of Dermatology, Chung-Ang University Hospital, Seoul 06973, Korea; drseo@hanafos.com

**Keywords:** atopic dermatitis, dupilumab, Patient Oriented Eczema Measure (POEM), female gender, lactate dehydrogenase (LDH), eosinophilia

## Abstract

Among biological agents for the treatment of atopic dermatitis (AD), dupilumab is a front-runner. Although many studies have been conducted on the real-world use of dupilumab, the sample size is often small and data is primarily on Western people. Therefore, we investigated the efficacy and safety of dupilumab in patients with moderate-to-severe AD in Korea. All patients with moderate-to-severe AD treated with dupilumab from September 2018 to June 2019 in this institution were included and analyzed by medical records. They were evaluated using the Eczema Area and Severity Index (EASI), Numerical Rating Scale (NRS), Patient Oriented Eczema Measure (POEM), and Dermatology Quality of Life Index (DLQI), respectively on admission, after two weeks (only EASI and NRS) and after 16 weeks. Laboratory tests were measured before and 16 weeks after treatment. A total of 101 patients were included. All efficacy tools showed a significant decrease after 16 weeks; EASI 77.4%, NRS 70.0%, POEM 60.7%, and DLQI 65.0%. EASI was characterized by a marked improvement of 51.5% in just two weeks. The treatment response was not significantly different according to the interval of treatment. Elevated Lactate Dehydrogenase (LDH) at 16 weeks was associated with poor treatment response. Moreover, a high eosinophil count was related to a lower change in EASI and POEM. In the correlation analysis, EASI was not correlated to DLQI before treatment. For changes after 16 weeks, POEM showed the highest correlation with DLQI. (R = 0.66, *p* < 0.001) In the additional analysis for factors affecting treatment response, the female gender was associated with good treatment response. (odds ratio = 5.4, *p* = 0.04) Adverse events from treatment included facial erythema (9.9%) and conjunctivitis (5.0%). Overall, it was confirmed that the efficacy of dupilumab in the real-world is similar to that of the existing clinical trials. We suggest that POEM is a useful tool for identifying the quality of life. The female gender was associated with a good treatment response. Both an elevated LDH and a high eosinophil count could be a therapeutic biomarker. Further research will be needed for a long-term period.

## 1. Introduction

Dupilumab is the first approved biologic agent for atopic dermatitis (AD). It acts by inhibiting the differentiation of naive T-cells into T helper (Th) 2 cells and inhibits the proliferation of Th 2 cells by blocking interleukin (IL)-4 and IL-13 simultaneously [1,2,3]. IL-4 and IL-13 are involved in the upstream initiation and clonal expansion of the Th2 reaction and play an essential role in the downstream Th2 reaction as Th2 effector cytokines. Therefore, theoretically, dupilumab might be an efficacious therapeutic agent.

Many real-world studies of dupilumab have been conducted, however, most studies [4,5,6,7] have used small sample sizes, and have usually been conducted using a Western sample. Therefore, to identify the efficacy and safety of dupilumab in Korea, we evaluated the real-world use of dupilumab in patients with moderate-to-severe AD in Korea. This study included the largest sample size from a single institution in Korea, and evaluated various efficacy measures during the treatment, although it was not a clinical study. In addition, we performed correlation analysis between efficacy measures to see the correlation between them, and identified the variables affecting the treatment outcomes.

## 2. Methods

### 2.1. Study Design and Data Collection

All patients with moderate-to-severe AD treated with dupilumab between September 2018 and June 2019 were included, and their cases were retrospectively analyzed using medical records. This study was approved by the Institutional Review Board (IRB) of National Medical Center and conducted in accordance with research ethics. AD was diagnosed by a dermatologist associated with our clinic according to the revised Hanifin and Rajka criteria [8]. Inclusion criteria were age >18 years and an Eczema Area and Severity Index (EASI) score >16 to include moderate-to-severe AD. The basic treatment regimen was treatment with dupilumab alone. If patients were treated with systemic steroid or cyclosporine before treatment of dupilumab at baseline, the systemic therapy was maintained to prevent a recurrence. At each visit, the dermatologist evaluated the status of AD. In the case of worsening or slower improvement than before, concomitant systemic treatment was maintained. In the case of improvement, it was gradually tapered, and in some cases, the concomitant treatment was discontinued. The dose of dupilumab was 600 mg at first administration and 300 mg during the subsequent visit. The interval between the doses was two weeks. However, there were some cases in which the interval was three, four, or more weeks depending on the patient’s financial status or distance from hospital; dupilumab is not covered by the National Health Insurance in Korea. As representative factors for AD, EASI, Numerical Rating Scale (NRS), Patient Oriented Eczema Measure (POEM), and Dermatology Quality of Life Index (DLQI) were measured before treatment and 16 weeks after the first dose of dupilumab [9,10,11,12]. EASI and NRS were also measured two weeks after the first dose. Laboratory tests such as those for immunoglobulin E (IgE), lactate dehydrogenase (LDH), and total eosinophil count (TEC) were conducted before and 16 weeks after the first dose.

### 2.2. Statistical Analysis

All data processing was performed using the R 3.5.2 version (Microsoft Corp., Redmond, WA, USA). Differences between the baseline and 16 weeks post-treatment measures were analyzed using a Wilcoxon signed rank test. Correlation analyses were performed to understand the correlations between efficacy measures. Also, the Wilcoxon rank-sum test and the Kruskal–Wallis test were performed to analyze the differences in efficacy measures based on the interval between doses and administration of concomitant treatment. The differences in response to treatment based on LDH and TEC levels at baseline and 16 weeks were also analyzed. Bonferroni correction was used for post-hoc analyses. The threshold of statistical significance was set at *p* < 0.05.

## 3. Results

### 3.1. Demographics

In total, 101 patients were enrolled (65 men and 36 women). Of these, 34.7% of patients had a family history of allergic diseases. Additionally, 77 patients (76.2%) had allergic diseases other than AD, of which, allergic rhinitis accounted for 67.5%, allergic conjunctivitis 18.2%, and asthma 14.3%. The antigens causing the allergy had been identified in 75 patients (74.3%): 57.3% were house dust mite and 22.7% were mold. Regarding previous treatment, 89 patients (88.1%) had been treated with topical steroids and 66 patients (65.4%) with topical calcineurin inhibitors; 66 patients (65.4%) had been treated with systemic steroids; 73 patients (72.3%) had taken systemic immunosuppressants, of which, 61 (82.4%) had taken cyclosporine; and 43 patients (42.6%) had received oriental medicine or a folk remedy (Table 1).

For the interval of dupilumab treatment, 50 patients (49.5%) maintained two weeks, and 29 patients (28.7%) took the doses at four-week intervals. There were 80 patients (79.2%) who took no concomitant therapy, while nine, seven, and five patients were on concomitant cyclosporine, methotrexate, and systemic steroid therapy, respectively (Table 2).

### 3.2. Efficacy of Treatment

#### 3.2.1. Baseline Values of Efficacy Measures

The median baseline values before the administration of dupilumab were EASI 29.0, NRS 8.0, POEM 24.0, and DLQI 23.0 (Table 3).

#### 3.2.2. Changes in EASI and NRS at 2 Weeks

At two weeks the decrease was 51.5% in EASI and 50.0% in NRS (*p* < 0.001; Table 3).

#### 3.2.3. Change in Efficacy Measures at 16 Weeks

All measures showed a significant decrease (*p* < 0.001), EASI by 77.4%, NRS by 70.0%, POEM by 60.7%, and DLQI by 65.0%. Patients who were only on dupilumab therapy without any concomitant treatment also showed a significant decrease in the efficacy measures (Table 3); this was done to see the effect of dupilumab alone.

#### 3.2.4. EASI 50 and EASI 75 at 16 Weeks

EASI 50 and EASI 75 mean the proportion of patients showing improvement of more than 50% and 75% in EASI, respectively. Two weeks after the first administration of dupilumab, the proportion of EASI 50 and EASI 75 was 51.4% and 2.8%, respectively. After 16 weeks, EASI 50 was 92.7%, and EASI 75 was 63.6%.

#### 3.2.5. Changes in Laboratory Tests at 16 Weeks

IgE, TEC, and LDH were measured at follow-up. At 16 weeks, only LDH was significantly reduced (in 24.1% of the patients; *p* = 0.010). However, there was no significant change in values in the dupilumab-only treatment group (Table 4).

#### 3.2.6. Relationship Between LDH or Eosinophil Level and Treatment Response

To identify the variables affecting treatment response, we analyzed the relationship between elevated LDH or eosinophilia and response to treatment. We found that in cases in which LDH was persistently elevated at 16 weeks, changes in NRS, POEM, and DLQI were significantly lower than those in the normal LDH group (Table 5). We also found that patients with eosinophilia (>500) and hypereosinophilia (>1500) at baseline showed a significantly smaller change in POEM and DLQI at 16 weeks than those without eosinophilia and hypereosinophilia. Moreover, patients with eosinophilia and hypereosinophilia at 16 weeks showed significantly smaller EASI and POEM changes than those without eosinophilia and hypereosinophilia (Table 6).

### 3.3. Subgroup Analysis

#### 3.3.1. Baseline Characteristics in Subgroups

Before performing subgroup analysis, the baseline status of each subgroup was identified to be able compare results to one another. Regarding treatment interval, there were no significant differences in EASI, NRS, POEM, and DLQI between the groups. In contrast, the concomitant therapy group showed higher EASI, NRS, POEM, and DLQI levels than the dupilumab-only group (Table 7).

#### 3.3.2. Differences in Efficacy Measures According to Dosage Interval

Most patients had a two-week interval, however, the two-week interval group’s efficacy measures were not significantly different from those of the other group with a longer interval.

#### 3.3.3. Differences in Efficacy Measures with Concomitant Therapy

We analyzed the differences in treatment response between patients concomitantly treated with systemic steroids, cyclosporine, or methotrexate, and those treated only dupilumab as the primary therapy. The change in EASI (*p* = 0.046), POEM (*p* = 0.020), and DLQI (*p* = 0.031) between the two groups at 16 weeks was significantly different. Patients in the dupilumab-only group showed a larger change than the concomitant treatment group.

### 3.4. Correlations Between Efficacy Measures

#### 3.4.1. Before Treatment

Correlation analysis was performed between the efficacy measures before the first dose of dupilumab. The results showed significant correlations between NRS and POEM (*R* = 0.54, *p* < 0.001), NRS and DLQI *(R* = 0.5, *p* < 0.001), POEM and DLQI (*R* = 0.45, *p* < 0.001), EASI and NRS (*R* = 0.26, *p* = 0.009), and EASI and POEM (*R* = 0.21, *p* = 0.042). The value of NRS was most related to that of DLQI; POEM was related, but EASI was not significantly related.

#### 3.4.2. Changes at 16 Weeks After the Treatment of Dupilumab

Correlation analyses between the changes in the values of efficacy measures was performed at 16 weeks after the first administration of dupilumab. Significant correlations in changes were observed between POEM and DLQI (*R* = 0.66, *p* < 0.001), NRS and POEM (*R* = 0.46, *p* = 0.001), NRS and DLQI (*R* = 0.43, *p* = 0.001), EASI and POEM (*R* = 0.4, *p* = 0.003), and EASI and DLQI (*R* = 0.39, *p* = 0.005). The measures related to changes in DLQI were POEM, NRS, and EASI, in order of significance. We also analyzed the correlation between changes in efficacy measures and laboratory tests. Only TEC showed a significant correlation with DLQI (*R* = 0.4, *p* = 0.015). Among the laboratory tests, TEC and LDH showed significant correlation (*R* = 0.59, *p* = 0.033).

### 3.5. Adverse Events After Treatment

Ten patients (9.9%) showed aggravation of erythema or newly developed erythema of the face after administration of dupilumab; among them, one patient was diagnosed with Systemic Lupus Erythematosus (SLE).

Five patients (5.0%) developed conjunctivitis after the administration of dupilumab. They were referred to ophthalmologists and showed improvement in conjunctivitis.

## 4. Discussion

This retrospective real-world study of dupilumab in Korea revealed that the efficacy of dupilumab in the real world is similar to that of the existing clinical trials. In addition, we identified the characteristics of moderate-to-severe AD patients in Korea and performed analyses on the correlations between efficacy measurements and variables affecting the treatment response.

In this study, the proportion of allergic conjunctivitis and asthma was lower than that of allergic rhinitis. This result contrasts previous clinical trials [13,14]. The low proportion of AD patients with asthma is distant from allergic march [15]. Therefore, large-scale cohort studies would be necessary to accurately understand allergic comorbidities in Korea.

The mean DLQI of our patients was higher than that of patients in previous clinical trials [3,13,14], because unlike in other countries, 42.6% of Koreans AD patients in this study received oriental or folk treatment, suggesting that this population is inclined to opt for alternative treatment for atopic dermatitis. The poor therapeutic effect [16] might be considered a factor affecting the quality of life.

In the study, the different treatment intervals could be a limitation, but we were able to compare the different interval groups in the treatment responses. Despite no difference in baseline status between the groups (shown in Table 7), there was no significant difference between the efficacy measures at different intervals. It is supposed that in cases showing good response to treatment, increasing the interval to four weeks or more could be considered during treatment. However, since our conclusions are based on clinical progress without drug pharmacokinetics, planned clinical trials are necessary.

We showed that the pre-treatment status of AD was worse in the concomitant group than in the dupilumab-only group (shown in Table 7). Since AD was already more severe in the concomitant group before the administration of dupilumab, the efficacy in the concomitant group was less than in the dupilumab-only group. Accordingly, concomitant treatment should be administered parallelly, and gradual tapering is required for preventing rebound effect.

**Figure 1:**
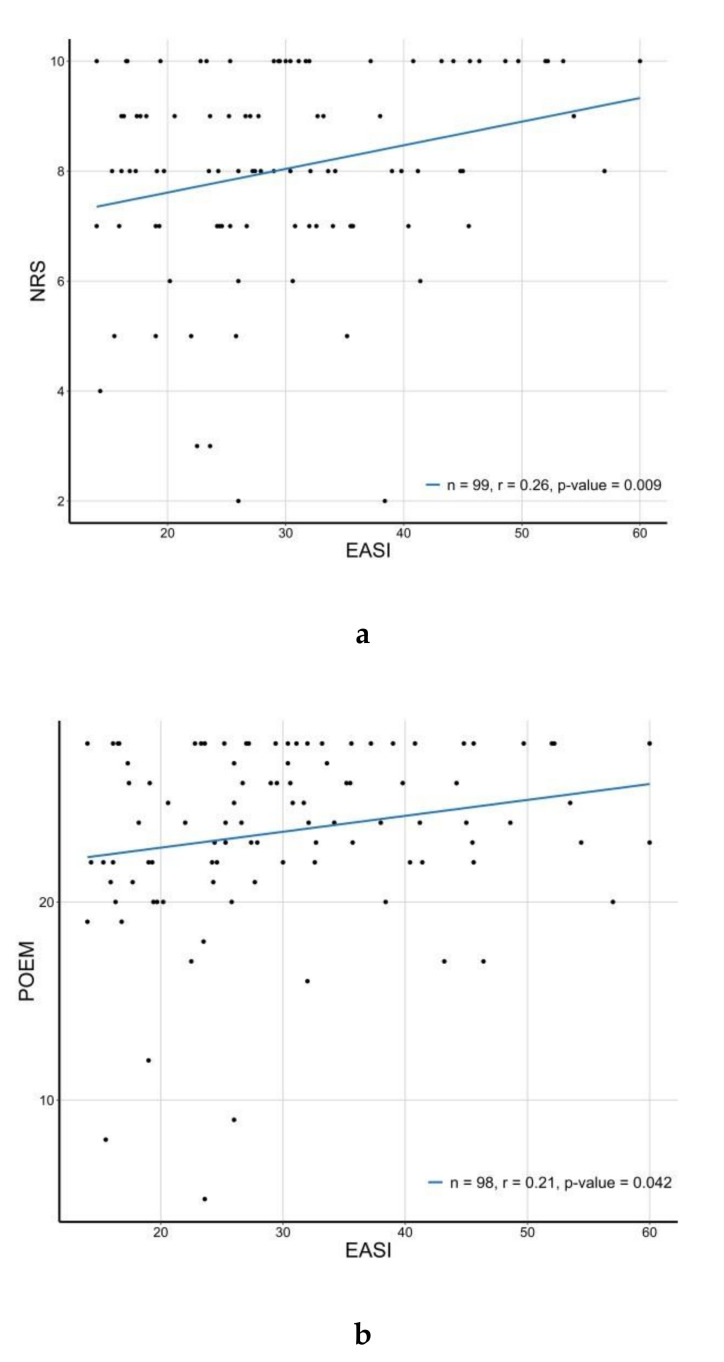
Correlation between (**a**) Eczema Area and Severity Index (EASI) and Numerical Rating Scale (NRS) (*R* = 0.26, *p* = 0.009), and (**b**) EASI and Patient Oriented Eczema Measure (POEM) (*R* = 0.21, *p* = 0.042) at baseline.

In the correlation analyses, NRS, POEM, and DLQI, which represent the patients’ subjective symptoms, showed significant correlations with each other. However, EASI, which may represent the objective symptom, was not significantly related to DLQI. This result suggests that the physical state of the lesion and the subjective symptoms might not be related. As shown in Figure 1a,b, despite a low EASI score, the subjective symptoms might be severe. It suggests that subjective symptoms should not be overlooked despite low EASI scores.

Regarding the changes for 16 weeks, POEM and DLQI showed the highest correlation, but EASI and DLQI showed the lowest correlation. It is different from a previous study on psoriasis [17], where a higher Psoriasis Area and Severity Index (PASI) correlated with a more significant change in DLQI. In psoriasis, the visible lesions may have a more significant impact on the quality of life than the subjective symptoms and go through the chronic course rather than the acute stage. In contrast, subjective symptoms, such as itching, have a more significant impact on the quality of life than the visible lesions do in the case of AD. Moreover, in addition to the chronic course, the symptoms of acute-stage lesions can greatly affect the patient. Due to the highest correlation with DLQI, we infer that POEM is particularly useful in evaluating the patient’s condition, expecially as POEM is easily done in a short time.

Not all patients who use dupilumab have a good prognosis; thus, much more attention is being directed at the factors affecting therapeutic effect. To identify the variables causing differences in response to treatment, we divided the study population into two groups based on the achievement of EASI 75. The only significant parameter was gender, with an odds ratio for women achieving EASI 75 equaling 5.4 (*p* = 0.04). It indicates that women are more likely to achieve EASI 75. This result is consistent with previously published real-world data in France [5].

There was no significant difference in the change in LDH after 16 weeks between the groups, but the elevation of LDH after 16 weeks was related to lower treatment response as indicated by subjective efficacy measures (Table 5). Although not related to EASI, this result is consistent with a recent real-world study of dupilumab [5], where LDH was associated with a therapeutic response. Therefore, LDH could be used as a biomarker for therapeutic response.

Another possible factor is eosinophil count; the EASI 75 achieving group showed lower TEC both at baseline and 16 weeks, and the patient with high eosinophil count at 16 weeks showed a lower change in EASI (Table 6). There are no laboratory findings related to hypereosinophilia in those with high TEC. This result indicates that a high eosinophil count might affect the treatment response, and a persistently high eosinophil count might be related to an inadequate response to treatment. In a previous study [18], there were some cases of eosinophilia after treatment with dupilumab.

Regarding adverse effects, facial erythema is a major concern associated with dupilumab in other countries [19,20], but the mechanism is not well understood. Facial erythema might be related to the worsening of existing AD lesions, withdrawal of systemic treatment, concomitant allergic contact dermatitis, or the adverse effects of dupilumab.

Conjunctivitis was reported in the clinical trials and has become a constant problem. The cause of dupilumab-associated conjunctivitis, as well as the relationship between its various pathogenetic mechanisms, are currently unclear, but possible hypotheses include inhibition of IL-4 and IL-13 signaling pathways involved in the development of atopic keratoconjunctivitis. Additionally, it appears to be aggravated in people with a history of allergic conjunctivitis. For the accurate diagnosis, symptoms such as itching of the eyelids and eyes or exacerbation of a periorbital facial-skin problem are important.

## 5. Conclusions

This study showed the efficacy of dupilumab similar to previous clinical trials, and that the POEM is helpful to represent symptoms of AD and the quality of life. Furthermore, elevated LDH and high eosinophil count might be related to inadequate response to dupilumab treatment. There was no significant difference in treatment response at different intervals, implying that the interval can be increased during treatment. Finally, this study would be beneficial for the understanding of dupilumab treatment.

## Figures and Tables

**Table 1:** Baseline demographics and history of treatment (*N* = 101).

Type	Variables	Mean (Min, Max) or Count (%)
Demographics	**Age, Years**	30.11 (18.00–50.00)
	**Gender**	
	Male	65 (64.36%)
	Female	36 (35.64%)
	**Occupation**	
	Yes	72 (71.29%)
	**Marriage**	
	Yes	22 (21.78%)
	**Disease Onset**	
	Adult exacerbation	13 (12.87%)
	Childhood	88 (87.13%)
	**Family History of Allergy**	
	Yes	35 (34.65%)
	**Allergy History**	77 (76.24%)
	Allergic conjunctivitis	14 (18.18%)
	Allergic rhinitis	52 (67.53%)
	Asthma	11 (14.29%)
	**Known Allergy**	75 (74.26%)
	House dust mite	43 (57.33%)
	Mold	17 (22.67%)
	Food	8 (10.67%)
	Cat	4 (5.33%)
	Dog	3 (4.00%)
	**Ocular Disease Telated to Past Treatment**	18 (17.82%)
	Cataract	6 (33.33%)
	Cataract (had operation)	7 (38.89%)
	Corneal transplantation	1 (5.56%)
	Glaucoma	2 (11.10%)
	Retinal detachment	2 (11.10%)
History of treatment	**Topical Treatment**	
	Topical steroid	89 (88.12%)
	Topical calcineurin inhibitor	66 (65.35%)
	**Systemic Treatment**	
	Systemic steroid	66 (65.35%)
	Immunosuppressant	73 (72.28%)
	Cyclosporine	61 (82.43%)
	Methotrexate	9 (12.16%)
	Mycophenolate mofetil	2 (2.70%)
	Azathioprine	1 (1.35%)
	Phototherapy	10 (9.90%)
	Oriental medicine	43 (42.57%)
	Folk remedy	6 (5.94%)

**Table 2:** Characteristics of treatment.

Dose Interval	*N* = 101	Combined Treatment	*N* = 101
2 weeks	50 (49.50%)	Cyclosporine	9 (8.91%)
3 weeks	5 (4.95%)	Methotrexate	7 (6.93%)
4 weeks	29 (28.71%)	Systemic steroid	5 (4.95%)
Increase of interval	16 (15.84%)	None (dupilumab-only)	80 (79.21%)
Decrease of interval	1 (0.99%)		

**Table 3:** Percent change in efficacy tools after two weeks and 16 weeks. All scores showed a significant decrease.

	Baseline	After 2 Weeks	*p*-Value **	After 16 Weeks	*p*-Value **
Total group					
EASI score (min–max)	29.0 (14.0–60.0)	15.1 (4.3–37.5)		7.5 (1.7–29.6)	
Median percent change ± IQR * in EASI		51.5 ± 24.9	<0.001	77.4 ± 12.5	<0.001
NRS score (min–max)	8.0 (2.0–10.0)	4.0 (0.0–7.0)		3.0 (1.0–7.0)	
Median percent change ± IQR * in NRS		50.0 ± 25.8	<0.001	70.0 ± 27.8	<0.001
POEM (min-max)	24.0 (5.0–28.0)			9.0 (1.0–24.0)	
Median percent change ± IQR * in POEM				60.7 ± 31.4	<0.001
DLQI (min-max)	23.0 (14.0–30.0)			9.0 (0.0–25.0)	
Median percent change ± IQR * in DLQI				65.0 ± 29.1	<0.001
Dupilumab-only group ***					
EASI score (min-max)	26.9 (14.0–60.0)	14.4 (4.3–36.6)		6.4 (1.7–29.6)	
Median percent change ± IQR * in EASI		51.7 ± 24.6	<0.001	77.9 ± 12.5	<0.001
NRS score (min-max)	8.0 (2.0–10.0)	3.0 (0.0–7.0)		2.0 (1.0–7.0)	
Median percent change ± IQR * in NRS		57.1 ± 30.6	<0.001	70.0 ± 17.8	<0.001
POEM (min-max)	24.0 (5.0–28.0)			9.0 (1.0–20.0)	
Median percent change ± IQR * in POEM				64.4 ± 27.0	<0.001
DLQI (min-max)	23.0 (14.0–30.0)			8.0 (0.0–25.0)	
Median percent change ± IQR * in DLQI				66.1 ± 27.0	<0.001

* The median percent change ± IQR was defined as the median of the percentage of change from baseline to 16 weeks. ** *p*-value calculated using the Wilcoxon signed-rank test. *** Dupilumab-only group without concomitant treatment. EASI: Eczema Area and Severity Index; NRS: Numerical Rating Scale; POEM: Patient-Oriented Eczema Measure; DLQI: Dermatology Life Quality Index; IQR: interquartile range.

**Table 4:** Change in laboratory test after 16weeks.

	Baseline	After 16 Weeks	*p*-Value **
Total group			
Serum total IgE (min–max)	316.5 (11.0–5000.0)	304.0 (6.0–2648.0)	
Median percent change ± IQR * in total IgE		3.48 ± 117.64 ****	0.981
Serum TEC (min–max)	672.0 (57.0–6066.0)	600.0 (82.0–6536.0)	
Median percent change ± IQR * in TEC		14.09 ± 84.80 ****	0.954
Serum LDH (min–max)	252.0 (130.0–491.0)	218.0 (132.0–385.0)	
Median percent change ± IQR * in LDH		24.14 ± 21.85	0.010
Dupilumab-only group ***			
Serum total Ig E (min–max)	368.0 (11.0–5000.0)	364.0 (52.0–2648.0)	
Median percent change ± IQR * in total IgE		5.01 ± 105.66 ****	0.636
Serum TEC (min–max)	563.0 (57.0–6066.0)	588.0 (82.0–3840.0)	
Median percent change ± IQR * in TEC		13.51 ± 77.75 ****	0.860
Serum LDH (min–max)	242.0 (130.0–392.0)	215.0 (132.0–348.0)	
Median percent change ± IQR * in LDH		21.27 ± 27.83 ****	0.098

* The median percent change ± IQR was defined as the median of the percentage of change from baseline to 16 weeks. ** *p*-value calculated using the Wilcoxon signed-rank test. *** Dupilumab-only group without concomitant treatment. **** Negative value means an increase compared with baseline. IgE: immunoglubulin E; TEC: total eosinophil count; LDH: lactate dehydrogenase; IQR: interquartile range.

**Table 5:** The difference of efficacy, according to LDH. Elevated LDH at 16 weeks showed inadequate treatment response in NRS, POEM, and DLQI.

	LDH at Baseline		LDH at 16 Weeks	
	<250	≥250	<250	≥250
Percent change of EASI after 16 weeks	77.77	65.09	76.83	77.33
*p*-value *		0.095		0.512
Percent change of NRS after 16 weeks	65.71	62.50	73.21	37.5
*p*-value *		0.640		<0.001
Percent change of POEM after 16 weeks	69.23	51.79	66.28	41.67
*p*-value *		0.182		0.014
Percent change of DLQI after 16 weeks	66.67	67.86	67.3	52.0
*p*-value *		0.764		0.021

* *p*-values were obtained using the Wilcoxon signed-rank test. LDH: Lactate Dehydrogenase; EASI: eczema area and severity index; NRS: numerical rating scale; POEM: patient-oriented eczema measure; DLQI: dermatology life quality index.

**Table 6:** The difference of efficacy, according to TEC. High TEC at baseline, showed a lower change in POEM after 16 weeks. High TEC at 16 weeks showed a lower change in EASI and POEM after 16 weeks.

	TEC at Baseline				TEC at 16 Weeks			
	<500	≥500	<1500	≥1500	<500	≥500	<1500	≥1500
Percent change of EASI after 16 weeks	76.96	76.04	77.78	73.49	77.67	69.27	78.00	73.25
*p*-value *		0.336		0.062		0.034		0.046
Percent change of NRS after 16 weeks	62.50	70.00	70.00	62.50	70.00	66.25	70.00	66.67
*p*-value *		0.806		0.994		0.836		0.455
Percent change of POEM after 16 weeks	69.23	53.97	64.71	37.50	64.71	33.94	69.51	53.57
*p*-value *		0.035		0.003		0.021		0.007
Percent change of DLQI after 16 weeks	71.42	56.83	67.26	50.00	65.51	53.26	67.33	56.52
*p*-value *		0.157		0.044		0.087		0.187

* *p*-values were obtained using the Wilcoxon signed-rank test. TEC: total eosinophil count; EASI: eczema area and severity index; NRS: numerical rating scale; POEM: patient-oriented eczema measure; DLQI: dermatology life quality index.

**Table 7:** Baseline characteristics in subgroup analysis.

	According to Treatment Interval		According to Concomitant Treatment	
	Interval of 2 Weeks	Interval Others	Dupilumab-Only Group	Concomitant Treatment Group
EASI score (min–max)	26.5 (14.0–60.0)	26.0 (14.0–60.0)	26.9 (14.0–60.0)	31.7 (15.9–60.0)
NRS score (min–max)	8.0 (2.0–10.0)	8.0 (2.0–10.0)	8.0 (2.0–10.0)	9.0 (2.0–10.0)
POEM (min–max)	25.5 (5.0–28.0)	23.5 (12.0–28.0)	24.0 (5.0–28.0)	25.0 (9.0–28.0)
DLQI (min–max)	23.0 (14.0–30.0)	23.0 (14.0–30.0)	23.0 (14.0–30.0)	26.0 (14.0–29.0)

## Trial Registration

Not applicable. We just retrospectively reviewed Electronic Medical Record (EMR).
